# Service utilization patterns and characteristics among clients of integrated supervised consumption sites in Toronto, Canada

**DOI:** 10.1186/s12954-022-00610-y

**Published:** 2022-03-29

**Authors:** Tanner Nassau, Gillian Kolla, Kate Mason, Shaun Hopkins, Paula Tookey, Elizabeth McLean, Dan Werb, Ayden Scheim

**Affiliations:** 1 Department of Epidemiology and Biostatistics, Dornsife School of Public Health, Drexel University, 3215 Market St, Nesbitt Hall, 5th Floor, Philadelphia, PA 19104 USA; 2 Canadian Institute for Substance Use Research, University of Victoria, Victoria, BC Canada; 3 South Riverdale Community Health Centre, Toronto, ON Canada; 4 The Works, Toronto Public Health, Toronto, ON Canada; 5 Centre On Drug Policy Evaluation, Li Ka Shing Knowledge Institute, St. Michael’s Hospital, Toronto, ON Canada; 6 Institute of Health Policy, Management and Evaluation, University of Toronto, Toronto, ON Canada; 7 Division of Infectious Diseases and Global Public Health, University of California San Diego School of Medicine, La Jolla, CA USA; 8 Department of Epidemiology and Biostatistics, Schulich School of Medicine and Dentistry, Western University, London, ON Canada

## Abstract

**Introduction:**

Supervised consumption services (SCS), intended to reduce morbidity and mortality among people who inject drugs, have been implemented in a variety of delivery models. We describe and compare access to and uptake of co-located and external services among clients accessing harm reduction-embedded (HR-embedded) and community health center-embedded (CHC-embedded) SCS models.

**Methods:**

Cross-sectional baseline data were collected between November 2018 and March 2020 as part of a cohort of people who inject drugs in Toronto, Canada designed to evaluate one HR-embedded and two CHC-embedded SCS. This analysis was restricted to clients who reported accessing these SCS more than once in the previous 6 months. Participants were classified as HR-embedded or CHC-embedded SCS clients based on self-reported usage patterns. Client characteristics, as well as access to onsite services and referral and uptake of external services, were compared by SCS model.

**Results:**

Among 469 SCS clients, 305 (65.0%) primarily used HR-embedded SCS and 164 (35.0%) primarily used CHC-embedded SCS. Compared to clients accessing CHC-embedded SCS, clients accessing HR-embedded SCS were somewhat younger (37.6 vs. 41.4, *p* < 0.001), more likely to report fentanyl as their primary injected drug (62.6% vs. 42.7%, *p* < 0.001), and visited SCS more often (49.5% vs. 25.6% ≥ daily, *p* < 0.001). HR-embedded SCS clients were more likely to access harm reduction services onsite compared to CHC-embedded SCS clients (94.8% vs. 89.6%, *p* = 0.04), while CHC-embedded SCS clients were more likely to access non-harm reduction services onsite (57.3% vs. 26.6%, *p* < 0.001). For external services, HR-embedded SCS clients were more likely to receive a referral (*p* = 0.03) but less likely to report referral uptake (*p* = 0.009).

**Conclusions:**

Clients accessing HR-embedded and CHC-embedded SCS were largely demographically similar but had different drug and SCS use patterns, with CHC-embedded SCS clients using the site less frequently. While clients of CHC-embedded SCS reported greater access to ancillary health services onsite, external service use remained moderate overall, underscoring the importance of co-location and support for clients with system navigation. Importantly, lack of capacity in services across the system may impact ability of staff to make referrals and/or the ability of clients to take up a referral.

## Introduction

Supervised consumption services (SCS) are intended to reduce drug-related harm and improve the health of people who inject drugs by providing medical and/or peer supervision while individuals consume pre-obtained drugs. The primary aims of SCS include prevention of overdose mortality and morbidity and infectious disease transmission [1, 2]. In Canada, SCS availability has been expanded since 2016 to address the country’s overdose crisis [3]. Most SCS also provide a low-barrier access point to a continuum of care, including primary care, substance use treatment, and social service programs, via service referrals or co-location [4].

SCS can be broadly categorized into three conceptual models of delivery: integrated, specialized, and mobile [5] (hospital-based and residential models are also growing in Canada). Whereas specialized SCS provide a focused range of on-site services related to sterile injection and overdose prevention, integrated SCS—generally situated within existing healthcare centers—provide a broader range of co-located services, from necessities such as food and clothing to opioid agonist treatment and primary healthcare. In practice, each SCS delivery model may be implemented with a significant degree of variability with respect to the range of services and delivery features. While an extensive body of research has demonstrated the impact of SCS on overdose and infectious disease transmission risks [6], less is known about how various service models, and particularly integrated SCS, impact client uptake of ancillary health and social services [4].

Existing research on the integration of tailored services for people who inject drugs has focused primarily on the combination of either opioid agonist treatment or needle and syringe programs (NSP) with infectious disease prevention or treatment [7–9]. For example, the vast majority of NSP in the United States report offering on-site HIV and/or HCV testing (89%) [10] and overdose education and naloxone distribution (94%) [11], and a survey of people who inject drugs accessing NSP in California found that about half received preventative services that were integrated into the NSP [12]. At the same time, the extent and impact of service integration may be limited due to stigma, criminalization of drug use, and the consequent need for robust privacy protections for people using SCS. Co-located services may not be fully integrated; for example, SCS in Toronto use a client database that is intentionally unlinked to electronic health records [13]. Further, SCS clients may not be interested in accessing co-located services for reasons including privacy concerns. In a qualitative study of SCS clients in Toronto, clients reported that integrated SCS made accessing health care and other social services easier, with benefits including not having to travel and the ability to find out about services they were previously unaware of [14]. However, some participants were concerned about a loss of privacy and anonymity related to integration of SCS within an existing health center.

Systemic barriers exist that may also impact the ability of SCS to provide clients with service referrals. Staff and participants at two SCS in Toronto noted several systemic issues, including the lack of shelter space for those experiencing homelessness, lack of treatment space for clients wishing to access addiction treatment services, funding insecurity for SCS, limited hours of operation, and the need for funding to support ongoing staff training [15].

Understanding the differences in client characteristics and service utilization between types of integrated SCS models is needed to enhance the impact of existing services and to provide insight for communities considering the implementation of varying SCS models. Drawing on baseline data from a cohort of people who inject drugs in Toronto, Canada, we therefore compared client characteristics, use of on-site services, and receipt and uptake of external referrals across two integrated models of SCS: a harm reduction program-embedded site (with on-site opioid agonist treatment) and sites embedded within comprehensive community health centers (CHCs).

## Methods

### Participants

As part of the Ontario Integrated Supervised Injection Services (OiSIS) cohort study in Toronto, Canada (OiSIS-Toronto), people who inject drugs completed baseline interviews from November 2018 to March 2020. Eligible participants were at least 18 years old, able to complete the interviewer-administered survey in English, reported having injected drugs non-medically in the previous 6 months, and were Toronto residents. Individuals were eligible to participate irrespective of their use of SCS, although for the purposes of this study only participants who reported SCS use were included. Study methods have been described elsewhere [13]. Briefly, participants were recruited at community health agencies, including at the agencies where SCS are located, and in the larger community through study staff, non-incentivized peer recruitment, and passive recruitment methods (posters and recruitment cards). Participants received a $30 honorarium for completing the baseline survey. Surveys were conducted at community health agencies, or at the research team offices if an alternative location was requested, by trained interviewers with lived experience.

### Integrated and specialized SCS

The OiSIS-Toronto cohort was designed to evaluate the first three SCS planned to open in Toronto, located at The Works (Toronto Public Health), Parkdale Queen West Community Health Centre, and South Riverdale Community Health Centre; and thus service referral and use questions focus on those sites. The three SCS included in this study opened between August 2017 and March 2018, operate under federal legal exemptions, and allow for injection, intranasal, and oral drug consumption [13]. SCS are staffed by medical and harm reduction workers, including individuals who have lived experience with drug use. At the time of this evaluation, there were a total of nine SCS open in Toronto. Participants who did not use any of the three SCS (*n* = 178), or who used them only once (*n* = 54), in the previous 6 months were excluded from these analyses. We categorized the three SCS based on their service model, with two of the sites being integrated within a community health center (CHC-embedded), and the third being integrated within a harm reduction program (HR-embedded). The CHC-embedded sites provide medical, mental health, and social care to vulnerable populations, and also have large onsite harm reduction programs that pre-date the opening of the SCS, while the HR-embedded site is a harm reduction program that includes a high-volume NSP, onsite nursing, and a small opioid agonist treatment clinic. Detailed descriptions of these SCS have been published previously [13].

### Measures

Participants were asked how often they accessed each SCS in the previous 6 months, with response options of more than once a day, once a day, every couple of days, once a week, every couple of weeks, or less than once a month, and were categorized as clients of CHC-embedded or HR-embedded SCS models. If a participant reported accessing both models, they were classified as either a CHC- or HR-embedded SCS client based on the site they reported using most frequently (given the geographic distance between sites, high-frequency use of both models was rare). Use of any other SCS open in Toronto did not impact classification.

Participants self-reported their age (continuous), sex at birth and gender (coded as cisgender woman, cisgender man, or transgender or gender diverse), race/ethnicity (Indigenous, non-Indigenous racialized, or white), and education (≤ high school, > high school). They also reported housing stability (any unstable housing [includes spending a night in the last 6 months in a place where people gather to use drugs, hotel/motel room, no fixed address, on the street, rooming or boarding house, shelter or hostel, or transitional housing] vs. stable housing), drug injected most often (fentanyl, heroin, prescription opioids, crystal methamphetamine, cocaine or crack/rock cocaine, other), and number of injections per day (all over the previous 6 months), as well as current receipt of medications for substance use disorders (yes vs. no).

Finally, participants were asked, separately for each of the three SCS, whether they used a range of on-site services (with a list specific to each site), and whether they (a) received referrals to and (b) actually used off-site services (with a common list across sites). On-site services were divided into two categories: harm reduction services (NSP, naloxone kits, overdose education and prevention, drug checking) and all other services (testing/vaccination; counselling; primary care; chiropody; dental; health promotion; HIV/HCV testing, support, and education; diabetes education; and group programming). External services were classified into six separate categories: healthcare, substance use treatment, social services, housing services, counselling, and other services.

### Statistical analysis

Descriptive statistics were stratified by primary use of CHC-embedded versus HR-embedded SCS. Chi-square and *t*-tests were used to compare client characteristics, use of onsite services (harm reduction vs. other), and referral and uptake of external services (across the six categories and overall). In addition, logistic regression models were fit to estimate the association between SCS model and (separately) referral receipt and uptake, adjusting for differences in client composition between models (age, primary injecting drug, SCS use frequency). Analyses were performed in SAS 9.4.

## Results

Overall, 469 participants reported using at least one of the three study SCS and were included in this analysis. These participants had a median age of 37.0 (IQR: 31.0–46.0), and were predominantly cisgender men (*n* = 293, 62.5%), white (*n* = 260, 55.4%), and unstably housed in the previous 6 months (*n* = 394, 84.0%). There were 305 (65.0%) classified as HR-embedded SCS clients, and 164 (35.0%) as CHC-embedded SCS clients. Participant characteristics by SCS type are shown in Table 1. CHC-embedded SCS clients were somewhat older than HR-embedded SCS clients (median age of 40.5 vs. 37, *p* < 0.001). Primary injection drug also was associated with SCS model type (*p* < 0.001), with fentanyl use being more common among HR-embedded SCS clients (62.6%) compared to CHC-embedded SCS clients (42.7%). HR-embedded SCS clients visited SCS much more frequently than CHC-embedded SCS clients, with 49.5% reporting using SCS at least once per day, versus 25.6% of CHC-embedded SCS clients (*p* < 0.001). There were no significant differences in gender, race/ethnicity, education, daily number of injections, or current receipt of substance use medications. Although the two groups had similar levels of housing instability overall, HR-embedded SCS clients were more likely to have slept outdoors in the previous 6 months (66.2% vs. 51.8%; *p* = 0.002; not shown).

**Table 1:** Participant characteristics by SCS type

	Type of SCS		*p* value
	HR-embedded *N* (Col %)	CHC-embedded *N* (Col %)	
Total	305 (43.5)	164 (23.4)	
Age, median (IQR)	37 (30–44)	40.5 (34–49.5)	< 0.001
Gender			0.732
Cisgender women	103 (34.0)	50 (30.5)	
Cisgender men	187 (61.7)	106 (64.6)	
Transgender or gender-diverse	13 (4.3)	8 (4.9)	
Race/ethnicity			0.968
Indigenous	98 (32.1)	53 (32.5)	
Racialized, non-indigenous	38 (12.5)	19 (11.7)	
White	169 (55.4)	91 (55.8)	
Education			0.948
High school or less	203 (66.8)	110 (67.1)	
Any post-secondary	101 (33.2)	54 (32.9)	
Housing**			0.532
Any unstable	260 (85.2)	134 (81.7)	
Stable	20 (6.6)	13 (7.9)	
Missing	25 (8.2)	17 (10.4)	
Drug injected most often**			< 0.001
Fentanyl	191 (62.6)	70 (42.7)	
Heroin	12 (3.9)	21 (12.8)	
Prescription opioids	9 (3.0)	21 (12.8)	
Crystal methamphetamine	61 (20.0)	22 (13.4)	
Cocaine or crack/rock cocaine	15 (4.9)	21 (12.8)	
Other	14 (4.6)	7 (4.3)	
Injections per day (Mean, SD)**	4.9 (4.2)	4.8 (3.7)	0.716
Currently receiving medication for substance use	113 (37.2)	63 (38.7)	0.753
Frequency of any SCS use**			< 0.001
Daily or more	151 (49.5)	42 (25.6)	
Once a week or more—less than daily	110 (36.1)	80 (48.8)	
Less than weekly	40 (13.1)	39 (23.8)	

Any unstable housing includes spending a night in the last six months in a place where people gather to use drugs, hotel/motel room, no fixed address, on the street, rooming or boarding house, shelter or hostel, or transitional housing

*HR* harm reduction, *CHC* community health center, *Col%* column percent, *IQR* Interquartile range

^*^0 missing age; 2 individuals missing gender; 1 individual was missing race/ethnicity; 1 individual missing education; 42 missing housing; 5 missing drug injected most often; 4 missing injections per day; 2 missing medication for substance use; 7 were missing frequency of any SCS use

^**^Over the previous six months

HR-embedded SCS clients were more likely to access harm reduction services onsite compared to CHC-embedded SCS clients (94.8% vs. 89.6%; *p* = 0.04), including 12.1% of HR-embedded SCS clients who accessed OAT onsite (not asked to CHC-embedded SCS clients as methadone is not offered onsite) (results not shown). Conversely, CHC-embedded SCS clients were more likely to access non-harm reduction services onsite compared to HR-embedded SCS clients (57.3% vs. 26.6%; *p* < 0.001). Distribution of harm reduction supplies (e.g. needle and syringe distribution, harm reduction supplies) was the most common onsite harm reduction service used (*n* = 287, 99.3% of HR-embedded SCS clients and *n* = 146, 89.0% of CHC-embedded SCS clients). The most common other type of onsite service used was testing and vaccination among HR-embedded SCS clients (*n* = 65, 21.3%), and group programming (e.g., wellness groups) among CHC-embedded SCS clients (*n* = 38, 23.2%).

Overall, HR-embedded SCS clients were more likely to be referred to external services compared to CHC-embedded SCS clients (36.1% vs. 26.2%; *p* = 0.03), but less likely to report uptake of external service referrals (59.1% vs. 81.4% of those referred; *p* = 0.009) (Table 2). CHC-embedded SCS clients had a 37% (OR: 0.63; 95% CI 0.41–0.96) lower unadjusted odds of receiving referrals to external services compared to HR-embedded SCS clients, which remained similar after adjusting for age, primary injecting drug, and SCS use frequency (aOR: 0.64; 95% CI 0.40–1.01) (data not shown). Among clients who received referrals, CHC-embedded SCS clients had greater unadjusted (OR: 3.03; 95% CI 1.29–7.14) and adjusted (aOR: 3.85; 95% CI 1.31–11.37) odds of accessing the services they were referred to, compared to HR-embedded SCS clients.

**Table 2:** Referral and access to external services by SCS type

	HR-embedded *N* (Col%)	CHC-embedded *N* (Col%)	*p* value
Total	305 (100)	164 (100)	
Any referral	110 (36.1)	43 (26.2)	0.03
Any uptake of referral*	65 (59.1)	35 (81.4)	0.009
Treatment/harm reduction			
Referral	39 (12.8)	12 (7.3)	0.07
Uptake*	22 (56.4)	8 (66.7)	0.53
Health care			
Referral	39 (12.8)	17 (10.4)	0.44
Uptake*	19 (48.7)	16 (94.1)	0.001
Social services			
Referral	26 (8.5)	12 (7.3)	0.65
Uptake*	12 (46.2)	11 (91.7)	0.008
Housing			
Referral	25 (8.2)	11 (6.7)	0.56
Uptake*	19 (76.0)	9 (81.8)	1.00
Counselling			
Referral	9 (3.0)	6 (3.7)	0.68
Uptake*	6 (66.7)	5 (83.3)	0.60
Other services			
Referral	17 (5.6)	1 (0.6)	0.008
Uptake*	10 (58.8)	1 (100.0)	1.00

*HR* harm reduction, *CHC* community health center, *Col%* column percent

^*^Among those who received a referral

Examining specific types of services (Table 2), uptake of referrals was greater among CHC-embedded SCS clients compared to HR-embedded SCS clients for health care (94.1% vs. 48.7%; *p* = 0.001) and social services (91.7% vs. 46.2%; *p* = 0.008). There were no differences in receipt or uptake of referrals by other types of services (except for ‘other’ services, which included food bank, legal services, education programs, etc.), but cell sizes were small.

## Discussion

In this study, we observed that clients accessing CHC-embedded SCS were largely demographically similar to clients accessing a HR-embedded SCS but were older and differed in their primary drugs used. Differences in the frequency of SCS use across the two service models may represent differences in the needs of the client populations and/or barriers related to how the models are structured. Compared to the CHC-embedded SCS locations, the HR-embedded SCS is located in an area with a higher concentration of people living and generating income outdoors and has longer operating hours (including being open on evenings and weekends). Previous qualitative research found that CHC-embedded SCS had several limitations including limited hours of operations [14]. Additionally, the geographic/neighborhood context of CHC- versus HR-embedded SCS may impact frequency of use. CHC-embedded SCS clients previously reported policing practices and the openness of a neighborhood’s drug scene as impacting their experience accessing SCS [16]. Although we cannot assume clients of the two models have the same baseline overdose risk, the finding that CHC-embedded clients have similar injection frequency but lower SCS coverage for injections may be a result of differences in operating hours, highlighting the importance of ensuring operating hours reflect client needs in order to improve service accessibility.

Although the vast majority of clients of both models accessed onsite harm reduction services, participants primarily using the HR-embedded site reported that they were more likely to do so. This is not unexpected, as the HR-embedded SCS is embedded within Toronto’s largest needle and syringe distribution program. A majority of CHC-embedded SCS clients used onsite health services not specific to drug use, as compared to about one-quarter of HR-embedded SCS clients, while the latter group was more likely to receive an external service referral. This may be due to the co-location of a wide range of services onsite with CHC-embedded SCS, which provides more opportunities for clients to receive onsite services and require fewer external referrals. While HR-embedded SCS clients received more external referrals than CHC-embedded clients, fewer HR-embedded SCS clients accessed provided referrals overall, and specifically to external health care and social services. This relationship held after adjusting for differences in client composition between the two models. HR-embedded SCS clients also demonstrated a greater drop-off between referral and access. This suggests that the wraparound care model and lower client volume [13] of CHC-embedded SCS may facilitate greater client engagement and case management opportunities by facility staff.

The level of referral and uptake of external services was 33% and 21% (65% uptake among those referred) of the study sample, respectively. Referral rates were remarkably similar to what was observed for an SCS in Sydney, Australia, where about 31% of clients received a service referral [17], and clients elsewhere have reported SCS as an important access point to additional services [15]. One possible reason for the observed rates of referral to external services may be that many clients use SCS infrequently, with infrequent clients having fewer opportunities to discuss their needs with staff or to receive support with accessing the referral. SCS staff may also forgo making referrals if they anticipate services will be unavailable due to a lack of capacity within the system and waitlists, or if they lack the appropriate resources, such as case management, given that making referrals can be a time-consuming process and is secondary to their primary aim of preventing overdose mortality. Additionally, observed referral and uptake rates may reflect the availability, or lack thereof, of necessary and accessible services for people who inject drugs. For instance, the limited uptake of housing services may reflect the systemic lack of affordable housing options in Toronto [18]. Finally, individuals may not receive referrals because they do not indicate to staff a need for any additional services.

There are several limitations to this study. First, as the data are cross-sectional, we cannot infer causality. Individuals may choose which type of SCS to use based on specific services they wish to access, and not vice versa. Second, SCS clients were not randomly sampled and may not be representative of the underlying client population. Third, service uptake and referral were self-reported and may underestimate the number of referrals actually provided by SCS staff. Further, questions concerning access to on-site services were site-specific and therefore not all services can be directly compared across all sites. For example, while the HR-embedded SCS has an on-site clinic offering opioid agonist treatment, the CHC-embedded SCS do offer buprenorphine/naloxone as part of primary care, however, only clients of the HR-embedded SCS were initially asked directly about on-site opioid agonist treatment. Finally, questions on external services pertained only to access via referral, rather than overall service utilization. Future analyses using linked healthcare administrative data will examine the relationship between CHC-embedded and HR-embedded SCS use and clinically-reported healthcare and opioid agonist treatment engagement [13].

Despite these limitations, the present study provides valuable information on the impact of integrating SCS within harm reduction programs and health care centers on access to a variety of services necessary for the health and wellbeing of people who inject drugs. It has been noted previously that there should be no one-size-fits-all approach to SCS [14], while others have recommended a variety of SCS service delivery models [15], and data presented here suggest that there are important differences in drug and SCS use patterns among clients who access CHC-embedded and HR-embedded SCS. Our finding that approximately one-third of clients reported referral, and nearly two-thirds of those referred reported uptake of services suggests that SCS play a critical role in connecting structurally vulnerable people who inject drugs with a continuum of care. While HR-embedded SCS clients had a higher referral rate to external services, those who were referred took up those referrals less often than clients of CHC-embedded SCS, suggesting that greater case management resources may be needed to support service access among clients of higher-volume SCS. There are many potential reasons for not accessing services, some of which (e.g., travel) can be overcome through an integrated service delivery model, while others (e.g., lack of capacity, trust) cannot. Future research is needed to understand SCS client perspectives on barriers and facilitators to use of co-located and referral services, as well as how access to these services changes over time through longitudinal study follow-up.

## Data Availability

The de-identified limited dataset used and analyzed during the current study is available from the corresponding author on reasonable request.

## References

[1] Marshall BD, Milloy MJ, Wood E, Montaner JS, Kerr T (2011). Reduction in overdose mortality after the opening of North America's first medically supervised safer injecting facility: a retrospective population-based study. Lancet.

[2] Kerr T, Tyndall M, Li K, Montaner J, Wood E (2005). Safer injection facility use and syringe sharing in injection drug users. Lancet.

[3] Kerr T, Mitra S, Kennedy MC, McNeil R (2017). Supervised injection facilities in Canada: past, present, and future. Harm Reduct J.

[4] Scheim A, Werb D (2018). Integrating supervised consumption into a continuum of care for people who use drugs. CMAJ.

[5] European Monitoring Centre for Drugs and Drug Addiction. Perspectives on drugs. Drug consumption rooms: an overview of provision and evidence. 2018.

[6] Potier C, Laprevote V, Dubois-Arber F, Cottencin O, Rolland B (2014). Supervised injection services: What has been demonstrated? A systematic literature review. Drug Alcohol Depend.

[7] Treloar C, Mao L, Wilson H (2016). Beyond equipment distribution in needle and syringe programmes: an exploratory analysis of blood-borne virus risk and other measures of client need. Harm Reduct J.

[8] Lucas GM, Mullen BA, Weidle PJ, Hader S, McCaul ME, Moore RD (2006). Directly administered antiretroviral therapy in methadone clinics is associated with improved HIV treatment outcomes, compared with outcomes among concurrent comparison groups. Clin Infect Dis.

[9] Cooke A, Saleem H, Mushi D, Mbwambo J, Hassan S, Lambdin BH (2017). Convenience without disclosure: a formative research study of a proposed integrated methadone and antiretroviral therapy service delivery model in Dar es Salaam, Tanzania. Addict Sci Clin Pract.

[10] Behrends CN, Nugent AV, Des Jarlais DC, Frimpong JA, Perlman DC, Schackman BR (2018). Availability of HIV and HCV on-site testing and treatment at syringe service programs in the United States. J Acquir Immune Defic Syndr.

[11] Lambdin BH, Bluthenthal RN, Wenger LD (2020). Overdose education and naloxone distribution within syringe service programs—United States, 2019. MMWR Morb Mortal Wkly Rep.

[12] Heinzerling KG, Kral AH, Flynn NM (2006). Unmet need for recommended preventive health services among clients of California syringe exchange programs: implications for quality improvement. Drug Alcohol Depend.

[13] Scheim AI, Sniderman R, Wang R (2021). The Ontario integrated supervised injection services cohort study of people who inject drugs in Toronto, Canada (OiSIS-Toronto): cohort profile. J Urban Health.

[14] Bardwell G, Strike C, Mitra S (2020). "That's a double-edged sword": exploring the integration of supervised consumption services within community health centres in Toronto, Canada. Health Place.

[15] Kolla G, Penn R, Long C (2019). Evaluation of the overdose prevention sites at street health and St. Stephen's community house.

[16] Bardwell G, Strike C, Altenberg J, Barnaby L, Kerr T (2019). Implementation context and the impact of policing on access to supervised consumption services in Toronto, Canada: a qualitative comparative analysis. Harm Reduct J.

[17] WHO (2010). Further evaluation of the medically supervised injecting centre during its extended trial period (2007–2011): final report.

[18] City of Toronto. HousingTO 2020–2030 action plan. 2019; https://www.toronto.ca/wp-content/uploads/2020/04/94f0-housing-to-2020-2030-action-plan-housing-secretariat.pdf.

